# Machine Learning in Health Promotion and Behavioral Change: Scoping Review

**DOI:** 10.2196/35831

**Published:** 2022-06-02

**Authors:** Yong Shian Goh, Jenna Qing Yun Ow Yong, Bernice Qian Hui Chee, Jonathan Han Loong Kuek, Cyrus Su Hui Ho

**Affiliations:** 1 Alice Lee Centre for Nursing Studies National University of Singapore Singapore Singapore; 2 Faculty of Arts and Social Sciences National University of Singapore Singapore Singapore; 3 Susan Wakil School of Nursing, Faculty of Medicine and Health The University of Sydney Sydney Australia; 4 Department of Psychological Medicine Yong Loo Lin School of Medicine National University of Singapore Singapore Singapore

**Keywords:** machine learning, health promotion, health behavioral changes, artificial intelligence

## Abstract

**Background:**

Despite health behavioral change interventions targeting modifiable lifestyle factors underlying chronic diseases, dropouts and nonadherence of individuals have remained high. The rapid development of machine learning (ML) in recent years, alongside its ability to provide readily available personalized experience for users, holds much potential for success in health promotion and behavioral change interventions.

**Objective:**

The aim of this paper is to provide an overview of the existing research on ML applications and harness their potential in health promotion and behavioral change interventions.

**Methods:**

A scoping review was conducted based on the 5-stage framework by Arksey and O’Malley and the PRISMA-ScR (Preferred Reporting Items for Systematic Reviews and Meta-Analyses for Scoping Reviews) guidelines. A total of 9 databases (the Cochrane Library, CINAHL, Embase, Ovid, ProQuest, PsycInfo, PubMed, Scopus, and Web of Science) were searched from inception to February 2021, without limits on the dates and types of publications. Studies were included in the review if they had incorporated ML in any health promotion or behavioral change interventions, had studied at least one group of participants, and had been published in English. Publication-related information (author, year, aim, and findings), area of health promotion, user data analyzed, type of ML used, challenges encountered, and future research were extracted from each study.

**Results:**

A total of 29 articles were included in this review. Three themes were generated, which are as follows: (1) enablers, which is the adoption of information technology for optimizing systemic operation; (2) challenges, which comprises the various hurdles and limitations presented in the articles; and (3) future directions, which explores prospective strategies in health promotion through ML.

**Conclusions:**

The challenges pertained to not only the time- and resource-consuming nature of ML-based applications, but also the burden on users for data input and the degree of personalization. Future works may consider designs that correspondingly mitigate these challenges in areas that receive limited attention, such as smoking and mental health.

## Introduction

Chronic diseases account substantially for morbidity [1] and mortality [2] worldwide. The occurrence of such diseases can be attributed to behavioral risk factors such as smoking, poor nutrition, alcohol consumption, and the lack of physical activity [3]. Despite the implementation of numerous lifestyle-based health behavioral interventions targeting modifiable risk factors such as obesity, stress management, and sedentary habits, dropouts and nonadherence of individuals to such recommendations have remained high [4]. Given the complex interplay [5] of factors such as financial and psychosocial, as well as vagueness of recommendations [6,7], it is challenging for one to understand the reasons underlying nonadherence. Of these factors, the psychosocial ones affecting adherence are manifold, where the difficulty is in changing lifestyles, attitudes, and beliefs of the individual coupled with feelings of guilt. Moreover, the feelings of hopelessness and isolation expressed by the individual further worsen by the lack of resources (eg, lack of support, food, time for behavioral changes, and treatment-related information). Against this background, it is evident that individuals face unique obstacles to their adherence to behavioral changes. To address such obstacles, behavioral change interventions need to be both multifaceted and personalized [6].

Machine learning (ML) techniques such as artificial intelligence (AI) and natural language processing over the past decade have resulted in advancements in learning algorithms, increased availability of online data, and low developmental costs [8]. The field of ML builds upon advanced statistical, computational, and probabilistic techniques to construct systems that automatically learn from data sets and require limited (ie, supervised) or no (ie, unsupervised) human input to yield accurate predictions and insights [9]. ML has been incorporated in various health processes such as detecting and diagnosing conditions [10], assessing and monitoring population health [11], providing prognoses and predicting treatment outcomes [12], and improving health research and clinical administration [13].

Alongside advances in mobile and wearable sensor technologies, AI monitoring systems, and telecare services [14], ML has gained popularity given its ability to analyze information from vast and complex data sets to provide readily available personalized experiences for users [8]. This ability to deliver tailored interventions may address the said problem of nonadherence [6], offering promising potential in health promotion and behavioral change interventions. Considering the rapid advances in ML over the last decade, this review aims to provide an overview of the existing research on machine learning applications and harness its potential in health promotion and behavioral change interventions.

## Methods

### Overview

Given the relative novelty of this area of research, a scoping review was undertaken to provide an overview of the literature. This review adopted the 5-stage framework by Arksey and O’Malley [15] and reported according to the PRISMA-ScR (Preferred Reporting Items for Systematic Reviews and Meta-Analyses for Scoping Reviews) guidelines [16], which is as follows: (1) identifying the research question; (2) identifying relevant studies; (3) selecting the studies; (4) charting the data; and (5) collating, summarizing, and reporting the results. As a scoping review aims to map the extent and nature of studies in the literature rather assessing their quality [15], the team did not perform a formal quality appraisal on the studies thus included.

### Identifying the Research Question

The main research question formulated to guide this review was as follows: “How have machine learning technologies been used as a strategy for health promotion and behavioral change interventions?”

### Identifying Suitable Studies

A search strategy was formulated to identify studies on ML techniques to promote behavioral changes and physical or mental health. A total of 9 databases were searched from inception to February 2021, aimed at encompassing not only multiple disciplines (Scopus and Web of Science), but also specific disciplines, including biomedical (the Cochrane Library, Embase, PubMed, Ovid, and ProQuest), nursing and allied health (CINAHL), and psychology (PsycInfo). To maximize the number of articles generated, no limits were applied to the dates and types of publications in order to maintain a comprehensive and updated search [17]. Relevant keywords and Medical Subject Headings (MeSH) terms were used, including “Learning, Machine,” “Behavior Control,” “Healthcare,” and “Mental Health” (Multimedia Appendix 1). Where appropriate, the keywords were truncated, and Boolean terms were added to maximize the retrieval of all relevant articles. End references of the studies were also hand searched for relevant articles [18].

### Selecting the Studies

Primary studies (including user preliminary testing or preliminary studies) were eligible for title and abstract screening if they had incorporated ML in promoting health or behavioral changes and were published in English (since the team lacked access to interpreters). Since this review aimed to examine real-life implications and experiences of incorporating ML techniques in health applications, articles were included only if they had studied at least a group of participants. Accordingly, those with no such reported results such as conference abstracts, proposals, and newspaper columns were excluded. Additionally, articles were excluded if they had examined other areas of health, such as the detection or diagnosis of conditions, population-health monitoring, prognosis and prediction of treatment outcomes, and research and clinical administration. Lastly, as ML techniques might overlap with statistical approaches [19], the 3 reviewers exercised discretion in determining those articles that had deployed ML techniques for inclusion.

All records retrieved from database searches were uploaded onto EndNote X9 (Clarivate Analytics), followed by the electronic removal of duplicates. During the screening of titles and abstracts, the remaining articles were independently reviewed by 2 reviewers (JOY and BC). Articles not meeting the eligibility criteria were removed, while those deemed suitable by at least one reviewer were downloaded for further examination. The opinion of a third reviewer (YSG) was sought, and disagreements between the reviewers were consensually resolved.

### Charting the Data

For each study, data extraction was conducted by one reviewer (JOY) and independently verified by a second (YSG). The following information was tabulated: author, year of publication, aim, main findings, area of health promotion, user data analyzed, type of ML used, challenges encountered, and future research. Such information served to inform the subsequent formation of themes in this review.

## Results

### Article Selection

**Figure 1:**
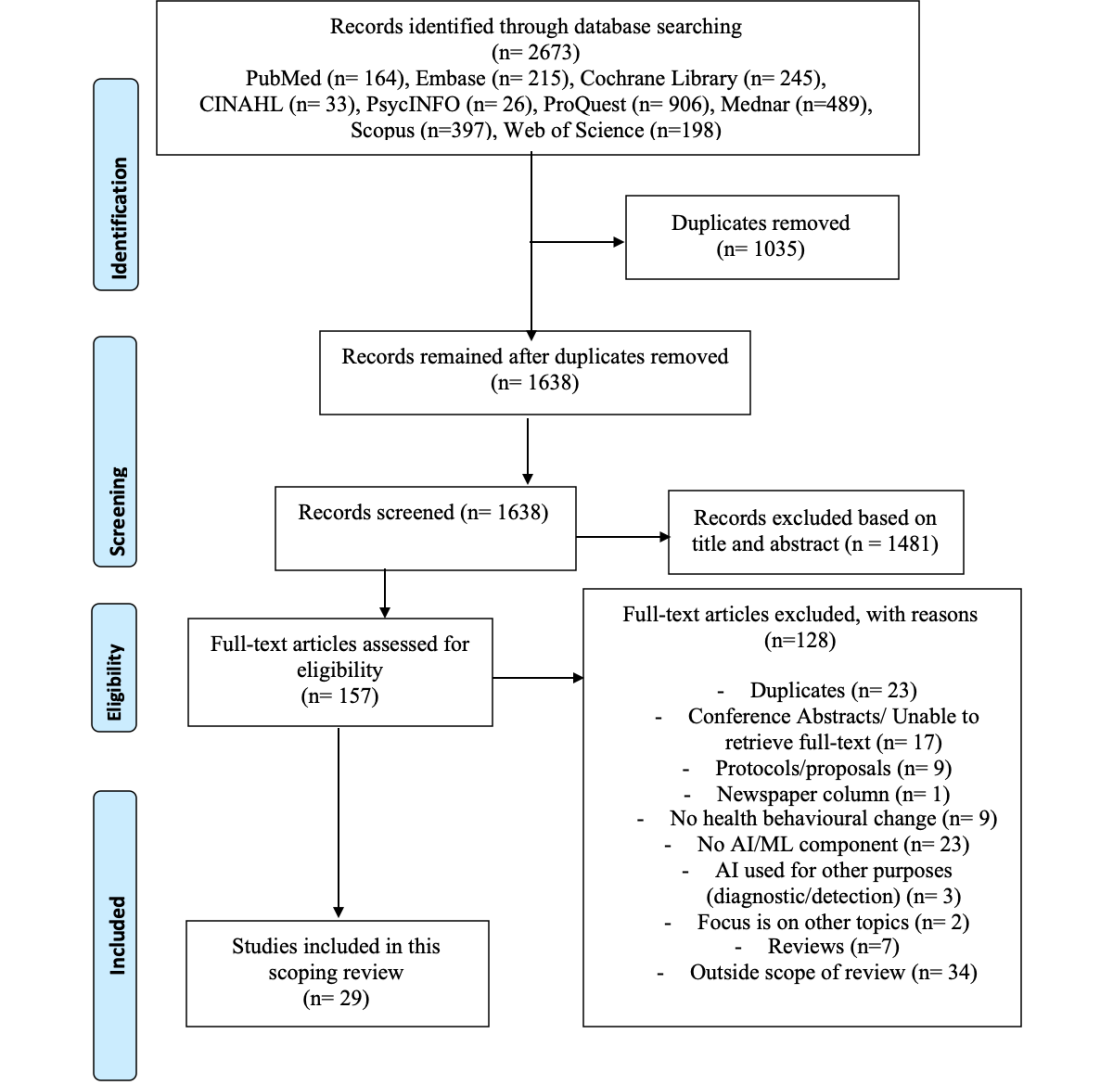
PRISMA (Preferred Reporting Items for Systematic Reviews and Meta-Analyses) flow diagram [16].

The literature search concluded in February 2021 and yielded 2673 search results. The removal of 1035 duplicates was followed by the screening of the titles and abstracts, during which another 1481 articles were removed. Of the remaining 157 articles included in full-text screening, 128 (82%) were removed with reasons such as duplicated articles, not using ML as main components, or not focusing on health behavioral change, leaving 29 (18.4%) for inclusion in this review (Figure 1).

### Characteristics of the Articles

The included articles were published between 2001 and 2020. The domains of health promotion presented in the articles were diverse, which are as follows: physical activity (9/29, 31%); smoking cessation (4/29, 14%); physical activity and dietary habits (4/29, 14%); stress detection or management (2/29, 7%); weight management (2/29, 7%); physical activity and sun-protection behavior (1/29, 3%); general self-health management (1/29, 3%); self-management for mental health (1/29, 3%); self-health management for hypertension (1/29, 3%); self-health management for sickle cell disease (1/29, 3%); help-seeking behavior for heart attack (1/29, 3%); diabetes prevention (1/29, 3%); and mindfulness meditation (1/29, 3%; Multimedia Appendix 2). Three themes emerged from the findings, which are as follows: (1) enablers, which it is the adoption of information technology for optimizing systemic operation; (2) challenges, which are the discussion of various hurdles and limitations presented in the articles; and (3) future directions, which explore prospective strategies in health promotion through ML.

### Themes

#### Theme 1: Enablers

Two key areas—the type of ML or AI technology used and data analyzed—have been identified as crucial to the implementation of the applications. Several studies used supervised ML techniques and algorithms in their designs [20-23]; in these studies, labelled data sets such as support vector machines [20], Markov logic network models [21], Naïve Bayes classifiers [23], and decision tree algorithms [22] were used to train algorithms for the purpose of classification or prediction. One study used unsupervised ML algorithms such as the Euclidean distance similarity algorithm [24], which identified hidden patterns in unlabeled data sets to recommend a best-fit message for users. Another study employed the multiarmed bandit model [25], a type of reinforcement ML technique that maximized cumulative reward to help users reach their behavioral goals. However, most studies either have not specified the type of ML or have stated only the use of rule-based algorithms [26-39].

Among these studies, several incorporated AI virtual agents such as embodied conversational agents or relational agents [22,31,34,38,40], while others incorporated chatbots in their designs [28,39,41,42]. The deployment of virtual agents allowed feedback and data output from ML algorithms to be presented to users and was typically designed to mimic human-like appearances or facial expressions. The data analyzed in the 29 studies included self-reports [20,24,26,29,32-37,39,43-48], GPS or wearable sensor data [20,23,25,29,31,36,39,45,46], application-generated data such as conversations with chatbots or users’ interactions in applications [21-24,27-29,31,34,35,38-42,45,46], and clinical data and health records [21,49].

#### Theme 2: Challenges

Two subthemes were generated to cluster the different challenges identified from the studies, which are (1) application- or ML-related challenges and (2) methodological challenges from current studies. Additionally, 4/29 (14%) studies [24,28,31,37] have not discussed any challenges and were thus excluded from this theme.

##### Application- or ML-Related challenges

Many studies have ascertained difficulties specific to their application or ML models. One such challenge was related to usability. In some studies, usability has been considered beforehand and thus would usually not complicate subsequent testing [28,44,45]. However, in 1/29 (3%) study targeting physical activity [29], problems with usability were noted because of space constraints in the participants’ homes and the need to switch between various digital systems in their homes. As the study involved older adults who might have had difficulties navigating complicated digital devices, the system’s usability warranted improvement.

Another challenge concerned the degree of personalization in health-behavior applications, as underlined in 4/29 (14%) studies [25,27,45,47]. A lack of personalization resulted in application-generated suggestions that were not tailored to the user [25]; it also resulted in the repetition of questions [27]. In addition, overpersonalization, intruding into the individual’s personal life, might breach confidentiality and privacy [45]. A similar challenge was the degree of automation in the applications, as highlighted by Block et al [49] and Traficante [47]. While the pilot test of their intervention demonstrated success in reducing diabetes-related factors, they suggested the caveat that the addition of human support might be more beneficial for specific users. Thus, the degrees of personalization and of automation in these health-behavior applications warrant prudent consideration of the balance between maximizing user benefits and minimizing drawbacks.

The accuracy of self-reported outcome measurements represented another hurdle, as reflected in 6/29 (21%) studies [34,35,39,43,44,48]. While some adopted a mixture of objective and self-reported outcomes [35,39,44], others collected only self-reported ones [34,43,48]. Such outcomes typically attempted to characterize health behaviors that were difficult to quantify, such as physical activity and dietary intake, for which the accuracy might thus be of concern. Furthermore, 1/29 (3%) study [20] reported the obtrusiveness of body sensors as a downside for its objective outcome measurements; to address this, the authors reduced the number of such sensors and monitored only the participants’ computer- and posture-based behavioral patterns.

Challenges also arose from specific components of the system, such as robot-programming [27], training ML models used in the applications [40,42], and tracking users’ lifestyles [32,36]. In robot-programming, the principal difficulty lay in ensuring human-like attributes in various robotic modules [27]. Such aspects included speech (intonation and speed), appearance (breathing, fidgeting, eye color, and face tracking), and fluency of interactions with users (comprehension of speech) [27]. Additionally, certain human aspects such as summarizing and reflecting upon the subjects’ meanings could not be replicated by the robot [27].

In training ML models used in the applications, the principal difficulty lay in the need for substantial input, as identified in 2/29 (7%) studies [40,42]. In both studies, their natural language models enabled chatbots or virtual coaches to comprehend users’ utterances and to respond appropriately. Given the prerequisite of considerable training for conversational fluency, users might be frustrated with the inability of the models to understand them [40]. To mitigate this, multiple-choice–based options, which were more effective than free-form natural language input, were added [40].

Lastly, in tracking users’ lifestyles, the principal difficulty lies in the inconvenience for the users, as highlighted in 2/29 (7%) studies [32,36]. The use of ecological momentary assessment with repeated logging to obtain the users’ data (eg, physical activity and dietary intake), was found to be both time-consuming and inconvenient [32]. Thus, Maimone et al [32] connected their tracking system to wearable devices with sensors and external functions (eg, weather forecasting and maps of bus stops). This not only minimized the burden on the users, but also added contextualized details to their input. Rahmani et al [36] took a step further by creating a personal chronicle (“Personicle”) of the users’ daily activities, integrating data from multiple streams such as sensor data, smartphone apps (eg, calendar, to-do lists, and social media), and ambient sounds.

##### Methodological Challenges From Current Studies

Apart from application- or ML-related challenges, methodological ones were also encountered. Of the 25 reviewed studies, 15 (60%) [21-23,29,33,35,38,39,41,43-48] cited challenges such as restricted generalizability due to small sample sizes, limited data collection, inadequate demographic representation, and the choice of a laboratory setting (ie, lack of ecological validity). Others included short testing periods [22,25,34,49] and limited outcomes due to the use of unrefined prototypes or algorithms [26,35,41,45,46]. These challenges were unsurprising since many of the included works were pilot, feasibility, or usability studies. Developers were often faced with limited resources, resulting in small-scale investigations with prototypes either of low fidelity or pending refinement. In such studies, late-stage participants tended to have better experiences since the cumulative addition of user data would enable both ongoing adjustments by the developers and continuous systemic improvements by the ML algorithms [41].

Additionally, difficulties were observed in ensuring the quality of collected data [20,39], in evaluative comparisons due to the lack of a control group [39,43,44,48], and in determining causality [39]. In ensuring data quality, the main difficulty pertained to errors in data input such as sensor failures or transmission faults [20] and in data entry by users [39]. For example, in the study by Stein and Brooks [39], data of physical activity collected through sensors could be altered by the users while data of dietary intake were manually entered into the app by them. Such data collection might have compromised the data quality when incomplete or inaccurate input occurred due to the users’ oversight. Lastly, the difficulty in determining causality has been reported by Stein and Brooks [39] due to the potential presence of confounding factors such as the subjects’ simultaneous engagement in other weight-loss interventions.

#### Theme 3: Future Directions

This review has provided valuable insights into ML-related research, including improvements on how ML-based technologies were being proposed for future health-promotion applications. The majority of the studies [20-22,24-29,31-34,36-49] have suggested the effectiveness of ML in health promotion and behavioral changes. However, some studies were unable to provide any statistical differences in their outcomes when comparing ML-based and typical applications [23,35]. Therefore, to address some of the said challenges and, accordingly, to present areas for development for future works, the following two subthemes were generated: (1) future studies and (2) future application of ML. Two studies [24,28] have not provided suggestions for any future research, which resulted in their exclusion from this theme.

##### Future Studies

This subtheme examines two areas that may be addressed—methodological designs and potential areas of research—to inform future studies on ML. In terms of methodological designs, many studies have underscored the need for longitudinal investigations to allow for more data collection for ML models and to determine whether the intervention-induced behavioral changes are sustained over prolonged periods [20,25,30,33,44,49]. This is especially true in order to rule out the novelty effect of using ML as an intervention for health behavioral change. Furthermore, additional buffer time was recommended for participants to accustom themselves to the app before the study [27]. Additionally, potential confounding variables should be accounted for by screening participants beforehand [39] and by broadening demographic representation, especially for age, ethnicity, and socioeconomic status [26,27,33,38,39,41,48,49]. Lastly, all stakeholders’ perspectives should be considered to holistically appraise the acceptability and usability of the application [41,47].

In terms of potential areas of research, suggestions for future studies are numerous. Studies could consider examining the effects of various measures, such as the following: notification content and purpose on users’ receptivity and response rates [23]; complex graphics such as games and chatbots in users’ engagement during behavioral changes [46]; and the use of digital services as active participants in users’ interactions [46]. Furthermore, research may be undertaken for tailoring variables to address smokers in the precontemplation stage [37], for comparing a simultaneous intervening mode with a sequential mode for physical activity and fat-intake interventions [43], for examining impacts of the emotivity of virtual agents [22], and for adding the element of user control to ML-generated suggestions [25]. Lastly, studies could also consider testing their app on other health behaviors [21,23,26,42].

##### Future Application of ML

Apart from addressing the identified challenges, several opportunities for future works have been highlighted for further developing and refining ML in health promotion and behavioral changes. Firstly, given the challenges of users’ inconvenience and human- and sensor-related errors [20,34,35,39,43,44,48], effective data-input modalities that require minimal users’ effort and encourage continuous engagement warrant greater attention [32]. Future works may accordingly consider developing systems that detect sensor failures [20] and those that account for erroneous self-reported measures [34]. Secondly, validated outcome measures and surveys have been found to exhibit low sensitivity in detecting subtle day-to-day behavioral changes, as exemplified by the short-term interventions in one study [34]. Future works may accordingly consider further examining the effectiveness of these interventions. Thirdly, ML-based applications offer the potential to be integrated into health care delivery systems [21]. However, care must be taken to ensure the reliability and safety of the information thus provided to users [21]. Lastly, Kulyk et al [31] have emphasized the need for guidelines and standardization for evaluating health technologies. They suggested multidisciplinary approaches and independent evaluators to examine the effects of interventions at all developmental stages of the app, not only to encourage continued engagement and motivation, but also to meet the target users’ needs.

## Discussion

### Principal Findings

This review has provided an insightful overview of the use of ML technologies in health promotion and behavioral changes, alongside a discussion of the challenges and potential opportunities for future works. Barring 4 studies [22,37,43,47], most others have been conducted over the past decade, indicating a recent growing interest in this topic. Additionally, the majority of the interventions in this review involved physical activity, whereas those specific to certain illness or those targeting smoking or mental health were relatively scarce. Hence, such interventions involving physical activity may be of interest for future works.

One noteworthy finding across the reviewed studies was the amount of time required to develop their ML-based applications. Some of the studies [20-25] specified the methods employed, including supervised ML, unsupervised ML, and reinforcement ML techniques. These differed in their use of supervised (labelled) data sets, which needed additional human intervention, and unsupervised (unlabeled) data sets. Unsupervised ML, coupled with the incorporation of virtual agents, embodied conversational agents, and human-like robots, required substantial time and resources to program natural language processing and to train the ML models [27,40,42]. Given the need for such length of time, the methodological implication was that most of the reviewed studies involved prototype research and pilot studies.

Furthermore, the complexity and comprehensiveness of ML-based applications grew with increasing amounts of data fed to their algorithms. The practical implication was that the experience differed between early-stage participants (who might experience a more rudimentary system) and their late-stage counterparts (who experienced a more refined one) [41]. These findings indicated that, to appraise the impacts of such ML-based applications more prudently and confidently, more studies examining them in a later, more mature, developmental stage would be warranted. Accordingly, developers might consider structured data input such as multiple-choice–based options [40] to aid the ML systems during the training of the application to refine and improve the accuracy of the feedback. These unique aspects of ML-based applications would deserve consideration in future studies incorporating ML techniques in their designs.

Another notable finding concerned hurdles in the design of the studies that might compromise their results, namely the lack of control groups [39,43,44,48], limited periods or samples for data collection [21-23,29,33,35,38,39,41,43-48], and potential confounders such as participation in other similar interventions [39]. To improve study designs and minimize the risk of confounders, the deployment of a waiting list control group and the disclosure of participation in other similar interventions should be instituted as part of the study methodology. Additionally, for short-term pilot and prototype studies with limited data collection, follow-up longitudinal studies might provide not only more data that would benefit the training of the ML system, but also insights into potential lasting effects of the intervention. Lastly, against such a background of these hurdles, health technologies would benefit from a set of guidelines and standardization, as advocated by Kulyk et al [31], for constant evaluation at all developmental stages to maximize the users’ potential to meet the target health behaviors.

Two other aspects merited discussion, which are as follows: (1) the burden on users to provide accurate data for tracking their lifestyles through self-reporting and the use of sensors; and (2) the challenges surrounding personalization. Although systems detecting sensor failures [20] and erroneous self-reported measures [34] might enhance the accuracy of behavioral measurement outcomes, further work in this area of research would be needed, especially with the increase in sensor usage in recent years [50]. Moreover, methods to reduce sensor failures through hardware enhancements could improve the quality of data. Additionally, both under- and overpersonalization would lead to overly generic feedback for users and breaches of privacy; this aspect thus warranted developers’ prudent and sensitive handling to strike a balance between providing customization and upholding privacy. Taken together, the challenges identified by the reviewed studies have underscored the need for a design that is nonobtrusive, requires minimal users’ input, encourages continuous engagement, and enables personalization while not encroaching upon privacy. Alongside such efforts, greater investment in combining multiple ML and AI techniques for synergy should likewise be pursued.

### Limitations

This review offers a timely overview on the emerging field of ML in health technologies, alongside potential referential value for future works. Nonetheless, several limitations are noteworthy. Firstly, the findings may not be generalizable since this review has included studies in only English due to the lack of access to interpreters. Additionally, the predefined scope of this review meant that studies examining other areas of health, such as detecting and diagnosing medical conditions, were excluded. Secondly, despite the use of numerous combinations to attempt to encapsulate the concept of ML in our search strategy for this review, the wide variety of ML techniques, alongside their overlap with statistical approaches [19], might have resulted in an omission of eligible studies. Finally, despite the demonstrable viability of ML techniques in health promotion and behavioral changes and their potential for integration into health care systems, most of the reviewed works have been pilot studies or prototype research, for which more investigations would thus be warranted to determine the clinical utility of the interventions.

### Conclusions

This review has examined the use of ML in health promotion and behavioral changes by mapping its associated challenges and highlighting potential areas for future works. The findings have collectively demonstrated that the challenges pertained to not only the time- and resource-consuming nature of ML-based applications, but also problems such as the burden on users for data input and the degree of personalization. Future works may consider designs that correspondingly mitigate these challenges in areas such as mental health promotion where the use of ML remains limited.

## Appendix

Multimedia Appendix 1 Keywords.

Multimedia Appendix 2 Characteristics of the included studies.

## Abbreviations

AI: artificial intelligence
MeSH: Medical Subject Headings
ML: machine learning
PRISMA-ScR: Preferred Reporting Items for Systematic Reviews and Meta-Analyses for Scoping Reviews
