# Supplementary material for: Machine Learning in Health Promotion and Behavioral Change: Scoping Review
Source: J Med Internet Res. 2022 Jun 2;24(6):e35831. doi: 10.2196/35831 (PMC9204568; doi:10.2196/35831)
Supplement: Multimedia Appendix 1 [file jmir_v24i6e35831_app1.docx]

**Multimedia Appendix 1: Keywords used in search**

| Concepts | Subject Headings | Keywords |
| --- | --- | --- |
| Machine Learning | “Learning, Machine”[Mesh]  “Transfer Learning”[Mesh]  “Learning, Transfer”[Mesh]  “Artificial Intelligence”[Mesh]  “AI”[Mesh]  “Intelligence, Artificial” [Mesh]  “Computational Intelligence”[Mesh]  “Intelligence, Computational”[Mesh]  “Machine Intelligence”[Mesh]  “Intelligence, Machine”[Mesh]  “Computer Vision System*”[Mesh]  “System*, Computer Vision”[Mesh]  “Deep Learning”[Mesh]  “Learning, Deep”[Mesh]  “Hierarchical Learning”[Mesh]  “Learning, Hierarchical”[Mesh]  “Big Data”[Mesh]  “Data Science”[Mesh]  “Algorithm*”[Mesh] | Machine learning OR learning, machine OR transfer, learning OR learning, transfer OR Artificial Intelligence OR AI OR Intelligence, Artificial OR Computational Intelligence OR Intelligence, Computational OR Machine Intelligence OR Intelligence, Machine OR Computer Vision System* OR System*, Computer Vision OR Deep Learning OR Learning, Deep OR Hierarchical Learning OR Learning, Hierarchical OR Big Data OR Data Science OR Algorithm* OR Human Computer Interaction |
| Behavioural Change | “Behavio*r Control”[Mesh]  “Control, Behavio*r”[Mesh]  “Behavio*ral Control”[Mesh]  “Control, Behavio*ral”[Mesh]  “Behavio*ral Manipulation”[Mesh]  “Manipulation, Behavio*ral”[Mesh] | Behavio*r Control OR Control, Behavio*r OR Behavio*ral Control OR Control, Behavio*ral OR Behavio*ral Manipulation OR Manipulation, Behavio*ral OR Behavio*r Change OR Behavio*ral Change OR Behavio*r Intervention* OR Behavio*r Change Intervention* |
| Health (general) | “Healthcare”[Mesh]  “Health Care”[Mesh] | Health OR Healthcare OR Health Care OR Health Intervention* OR e-Health OR Health-related |
| Mental Health | “Mental Health” [Mesh]  “Health, Mental” [Mesh]  “Mental Health Service*”[Mesh]  “Health Service*, Mental”[Mesh]  “Service*, Mental Health”[Mesh] | Mental Health OR Health, Mental OR Mental Health Service* OR Health Service*, Mental OR Service*, Mental Health OR Mental Healthcare OR Mental Health Care OR Mental Health Literacy OR Mental Health Awareness OR Mental Health Knowledge OR Mental Health Education |
