# Supplementary material for: Machine Learning in Health Promotion and Behavioral Change: Scoping Review
Source: J Med Internet Res. 2022 Jun 2;24(6):e35831. doi: 10.2196/35831 (PMC9204568; doi:10.2196/35831)
Supplement: Multimedia Appendix 2 [file jmir_v24i6e35831_app2.docx]

**Multimedia Appendix 2: Characteristics of included studies**

| ID | Author (Year) | Health promotion application | Purpose | Data analyzed | Machine learning/artificial intelligence technology application | Summary of relevant findings |
| --- | --- | --- | --- | --- | --- | --- |
| 1 | Ahsan et al. (2013) | Smoking cessation | To present a mobile health (mHealth) solution that leverages the Short Message Service (SMS) or text messaging feature of mobile devices to motivate behavior change among tobacco users. | User’s demographic profile, self-reports, user behaviour | User-based recommender engine algorithms (Euclidean Distance Similarity/Pearson Correlation Similarity/Log Likelihood Similarity) used to select the best-fit message from a list of motivational messages in database. | Preliminary usability testing revealed that 90% of 10 testers favoured the personalized messages while one user was indifferent to receiving a personalized message versus a randomly selected message of potentially less relevance. |
| 2 | Alberdi et al. (2018) | Stress detection | To build models that predict self-assessed stress and mental workload scores, as well as models that predict workload conditions based on physiological and behavior data. | Multiple body sensors and self-reported tests | Regression models (radial basis function (RBF) kernel support vector machines (SVM)) were built for the prediction of the self-reported stress and mental workload scores from data based on real office work settings.  Classification models (Naïve Bayes, linear SVM, AdaBoost and C4.5 tree algorithms) were employed to detect workload conditions and change in these conditions. | Results confirm the possibility of predicting the perceived stress and workload levels of office workers, as well as the objectively measured conditions they might be undergoing or the significant workload condition changes that they might be suffering from changes in unobtrusively collected smart office-based physiological and behavioral data.  Results also suggest that computer-use patterns together with body posture and movements are the best predictors for this purpose. |
| 3 | Brigham et al. (2014) | Smoking cessation | To develop a tailoring algorithm to depict the likelihood of successful cessation, given a range of intervention options from self-help to combinations of medications. | Self-report questionnaires | Computer-tailored algorithms | The algorithm prioritizes the content of a stop-smoking intervention individually for each user and indicates the potential effect of utilizing various stop-smoking medications and stop-smoking approaches.  The algorithm generates a set of probabilities that reflect the likelihood of successfully quitting, based on input of independent variables including age, tobacco dependence, quit attempt history, perceptions of stress and control, gender, body mass index, and proximity to another smoker. As those variables differ, the probability of success in quitting differs, resulting in variations in probability of successful quitting.  The algorithm predicts that even a daily smoker may be able to substantially improve the likelihood of quitting and staying quit both by using stop-smoking techniques and medications and by addressing emotional and cognitive issues that sustain smoking. |
| 4 | Chen et al. (2018) | Self-health management | To describe the design and evaluation of the The Design of Personalized Artificial Intelligence Diagnosis and the  Treatment of Health Management Systems Simulating the Role of General  Practitioners (AIHMS) that assists in providing tailored interventions to enhance health related behavioral changes. | Patients’ clinical data, wearable device monitoring data, and user-generated platform data | Classical supervised learning tasks for information extraction.  Markov logic network models constructed to provide patients with specific diagnoses and treatment services. | The results of preliminary system evaluations indicate that the AIHMS can reduce the patients’ compliance discontinuation rate and move more patients forward to the stage where discontinuation is less likely to happen.  The findings suggest that the integration of behavioral theories and the AIHMS can make a significant contribution to healthcare delivery for the public. |
| 5 | Jimison et al. (2015) | Physical activity | The focus of this paper is on the coaching approach to using health behaviour change and computational modelling to delivering an interactive video system for remote coaching of older adults in the home. | Health status, preferences, health behaviour change variables, multiple sensors, computer interactions, medications, phone usage, contact switches. | User models that incorporate dynamically varying behaviour change variables with algorithms that trigger just-in-time feedback and tailored coaching messages provide a framework for making health interventions more effective. | Results from pilot study demonstrates ability to maintain adherence to the exercise goals for most participants. However, there were many usability issues in initiating the program.  Remote coaching technology paired with new developments in interactive video offerings provide a promising approach for delivering much needed physical exercise health interventions. |
| 6 | Kulyk et al. (2014) | Physical activity | This paper presents new insights and general guidelines for design and evaluation of lifestyle change support systems that uses personalized virtual coaching.  The first field study focuses on design and evaluation of a mobile physical activity coaching system.  The second user study focuses on design factors that influence the attitudes of high-risk adolescents towards virtual coaching in mobile eHealth applications and social media. | Activity sensor, mobile applications, software | Rule-based digital coaching system with centralized architecture (Smarcos system) to provide reminders to connect the activity monitor to upload data, send a motivating message when activity is less than the target or an overview of daily, weekly and monthly scores.  Responsive animated Embodied Conversational Agents (ECAs) used for the presentation of feedback on a mobile app. The system can produce ECA behaviors (eye blink, eye gaze, head movements, lip sync with natural speech and facial expressions) specified in the Behavior Markup Language (BML). The BML contains the text to be pronounced by the ECA as well as the non-verbal embodiments | User studies showed positive attitude towards the use of a virtual coach for lifestyle change support.  Several stimulating persuasive features were identified in both studies:  1. Visual appeal is a crucial design factor as it determines whether a user is will get motivated to start with the lifestyle behavior change program. Various visual aids and user-centered design methods can ensure that visual representations of the interface are appealing to the target user group.  2. Effective coaching and tailored feedback in terms of its timing, content and interaction design are crucial elements in affecting behavioral change. Personal target goal has to be challenging and reachable, step by step, within a set period of time.  3. Users prefer to be in control of how, when (right timing) and on what device they want to receive personal feedback from a virtual coach.  4. There is a need to provide personalized (tailored) feedback: show progress towards target, adjust target, motivate, suggest actions, provide real time information. Fun element has to be integrated in user interface to make interaction with the coach more playful. |
| 7 | Rabbi et al. (2015) | Physical activity, dietary behaviour change | This study investigated the technical feasibility of implementing an automated feedback system (MyBehavior), the impact of the suggestions on user physical activity and eating behavior, and user perceptions of the automatically generated suggestions. | GPS sensor (in mobile phone), physical activity logs, food logs | Standard machine-learning, decision-making algorithm (multi-armed bandit (MAB)),  to generate personalized suggestions that ask users to either continue, avoid, or make small changes to existing behaviors to help users reach behavioral goals | In qualitative daily diary, interview, and survey data, users reported MyBehavior suggestions to be highly actionable  and stated that they intended to follow the suggestions.  MyBehavior users walked significantly more than the control group over the 3 weeks of the study.  Although some MyBehavior users chose lower-calorie foods, the between-group difference was not significant.  In a poststudy survey, users rated MyBehavior’s personalized suggestions more positively than the nonpersonalized, generic suggestions created by professionals. |
| 8 | Silverman et al. (2001) | Help-seeking behaviour | The goal of this research is to determine whether a computer-based training game (HEART-SENSE) can improve recognition of heart attack symptoms and shift behavioral issues so as to reduce pre-hospitalization delay in seeking treatment. | User's evaluations, decisions, actions in game | Decision tree algorithms, generic simulator package with animated pedagogical agent.  The agent senses what's going on in the game world to form her emotions and then to express them back to the user through her various effectors (voice, mouth, eyes, face, and posture and limb motions) to represent emotions ranging across ecstatic, happy, calm, nervous, dizzy, worried, and highly alarmed. | Initial results show that users of the game exhibit a significant shift in intention to call 9-1-1 and avoid delay, that multi-media versions of the game foster vividness and memory retention as well as a better understanding of both symptoms and of the need to manage time during a heart attack event.  User reaction data indicate the emotive pedagogical agent improves usability overall as well as learner performance during complex explanations. The emotive agents  are viewed as highly useful as companions and for entertainment that appears to keep user affect positive. |
| 9 | Monteiro-Guerra et al. (2020) | Physical activity | This study aimed to execute the design process and early prototype evaluation of a personalized PA coaching app  for posttreatment breast cancer survivors. In particular, the study explored a design combining behavioral theory and tailored coaching strategies. | Sensors (in mobile phone), user’s baseline level, progress, user’s perceived fatigue level (pre-session), user’s perceived session difficulty level (post-session). | Rule-based module that adjusts the step goal and training session objectives in line with the model of linear progression training  Virtual coach to provide general information, support, tips, targeted activity feedback, motivation, and summary report, | The results from prototype testing with users and experts were promising, with high scores for usability, quality, and behaviour change potential.  Several considerations can be taken from these evaluations to inform the future refinement of the prototype:  1. Further exploring engagement strategies, particularly related to entertainment, customization, and interactivity  2. Consider other behavior change techniques, such as providing the ability to export data from the app, to suggest restructuring social or physical environment, and to assist with distraction or avoidance.  3. Include resistance exercises in the activity program and app  4. Share experiences with close friends or family members  5. Enable data sharing with health care professionals. |
| 10 | Morrison et al. (2017) | Stress management | This exploratory mixed-methods study examined the potential impact of timing and frequency on notification response and usage of Healthy Mind, a smartphone-based stress management intervention. | Phone-based sensors (GPS, accelerometer, clock), app usage | Naïve Bayesian classifier algorithm established a relationship between specific contexts and likelihood of notification response: to determine timing and frequency of notification and adapting delivery to a user's current context (e.g. location). | This exploratory study suggests that tailoring the delivery of notifications based on users' current location and movement may not always encourage greater response rates or intervention usage in a naturalistic setting compared to sending notifications at assumed good times.  This study also suggests that sending frequent, daily notifications may enhance exposure to intervention content without deterring continued engagement. |
| 11 | Nurmi et al. (2020) | Physical activity | This study aimed to describe the theory- and evidence-based development of the Precious app and to examine how digitalized motivational interviewing using a smartphone app engages users in the behavior change process.  We aimed to determine if use of the Precious app elicits change talk in participants and how they perceive autonomy support in the app. | Heart rate variability sensor, activity bracelets, self-reports, user’s selections in app | Rule-based algorithms to tailor personalized suggestions. | The motivational interviewing features actively engaged participants in reflecting their outcome goals and reasons for activity, producing several types of change talk and very little sustain talk. This active engagement in self-reflection can be perceived as a proxy for engagement in the first steps of the behaviour change process. The fast pace with which users typically advanced through the app suggests that smartphone-based interventions may benefit from interactive functions instead of open questions and imagination tasks.  The feasibility interviews of the digitalized motivational interviewing features revealed that participants value personalization options, but the app also needs to clearly communicate how the information collected from users will be used for recommendations and tailoring and which choices users are able to change later.  Apps supporting behavior change need to engage users in the behavior change process. Feasibility tests with the Precious app suggest that this can be done by addressing users’ psychological needs and by supporting active self-reflection. |
| 12 | Persell et al. (2020) | Hypertension self-management (including diet, physical activity, medication adherence, blood pressure measurement, sleep, and stress management) | To investigate the effect of an artificial intelligence smartphone coaching app to promote home monitoring and hypertension-related behaviors on systolic blood pressure level compared with a blood pressure tracking app. | Blood pressure monitor, motion detector (in smartphone), user’s app data and responses | Algorithms to provide feedback and behavioral and educational coaching. | In a population with mild uncontrolled hypertension, a beta version of a smartphone coaching app to promote home monitoring and other behaviors associated with hypertension plus an home blood pressure monitor (HBPM) did not lower systolic blood pressures at 6 months compared with a tracking app and an HBPM. Intervention group participants had greater self-confidence in controlling blood pressure and may have increased exercise more compared with the control group. |
| 13 | Schumann et al. (2008) | Smoking cessation | This study examines tailored feedback letters of a smoking cessation intervention that is conceptually based on the transtheoretical model, from a content-based perspective. | Questionnaires | Rule-based algorithm to generate tailored, individualized feedback letters (scale scores are compared with cutoff scores, which indicate norms of scale scores stratified by stage of change. Then decision rules are applied to determine which feedback paragraphs have to be assembled into the interventions) | Results reveal an enormous theoretical variability with more than 1000 unique normative feedback letters and almost half a million unique ipsative feedback letters. The high theoretical variability is mirrored by a high empirical variability. The different unique 1^st^, 2^nd^, and 3^rd^ letters that are possible theoretically are necessary empirically.  Especially for contemplation and preparation, almost every single smoker needed a different letter, which means that they had different combinations of TTM-variables. This indicates great empirical interindividual variability, which is accounted for in the theoretical variability of the tailored TTM based intervention.  For smokers in contemplation and preparation, a higher theoretical number of unique letters is available than for smokers in precontemplation. The reason is that feedback for precontemplation is only based on 4 TTM-variables (decisional balance and 3 processes of change) while feedback for contemplation and preparation is based on 8 TTM variables (decisional balance, general and situation-specific self-efficacy, and 5 processes of change). Thus, the TTM offers greater theoretical variability for smokers who are further along in the quitting process. |
| 14 | Vandelanotte et al. (2007) | Physical activity and fat intake | This study aims to examine long-term efficacy of interactive computer-tailored physical activity and fat intake interventions, and evaluate their efficacy in a simultaneous or sequential implementation over 2 years. | Questionnaires | Expert system to select correct feedback messages from a large database. The highly individualized feedback consisted of three parts: a general introduction, normative feedback that related participants’ physical activity or fat intake to current recommendations, and tips and suggestions on how to increase physical activity or decrease fat intake. | The results indicate that most of the health behavioral changes induced by the interactive computer-tailored physical activity and fat intake interventions were maintained in participants that were available 2 years after the implementation of the interventions.  The intervention effects at 2 years were overall somewhat smaller than those that were reported at 6 months but still significant and rather substantial. These positive results were rather unexpected since participants were exposed to the intervention program only once. Both the simultaneous and sequential intervening modes were effective at 2-year follow-up. However, it appears that the sequential intervention overall resulted in better maintenance of intervention effects, whereas the opposite was true 6 months after baseline.  At 2-year follow-up the interventions resulted in a 10% and a 27% increase of participants that met public health recommendations for physical activity and fat intake, respectively.  Overall, the interventions were more effective for participants not meeting the public health recommendations at baseline. |
| 15 | Vandelanotte et al. (2018) | Physical activity | This study aimed to examine whether the effectiveness of a Web-based computer-tailored intervention could be improved by integrating Fitbit activity trackers. | Fitbit activity trackers, questionnaires, demographic information, body mass index, | If-then algorithms to automatically select personally relevant physical activity content from a database. | Linear mixed model analyses showed a significant increase in total weekly physical activity and moderate-to-vigorous physical activity in the Fitbit group compared with the non-Fitbit group at the 3-month follow-up.  The sitting time and BMI decreased more in the Fitbit group, but no significant group × time interaction effects were found. The physical activity advice acceptability and the website usability were consistently rated higher by participants in the Fitbit group.  Non-Fitbit group participants completed 2.9 modules, and Fitbit group participants completed 4.4 modules.  Hence, integrating physical activity trackers into a Web-based computer-tailored intervention significantly increased intervention effectiveness. |
| 16 | Issom et al. (2020) | Sickle-Cell Disease (SCD) self-management | This preliminary feasibility study examined patients’ perceived usefulness of the information provided by a chatbot we developed following patient-important requirements collected during our preliminary studies | App data | Fully automated coaching app/Chatbot (detailed information not available) | Results suggest that mHealth coaching apps could be used to promote the knowledge acquisition of recommended health behaviors related to the prevention of SCD main symptoms. |
| 17 | Almusharraf (2019) | Smoking cessation | The goal of this research is to design and deploy a computer-based conversational system  that engages cigarette smokers in a text-based MI conversation and observe the conversation's effect on their motivation to quit. | Demographic information, conversations with chatbot | Rule-based chatbot to generate conversation.  Natural Language Understanding (NLU) capabilities to inform the rules of the conversation | The Training and Intervention studies showed that subjects observed more positive than negative impact in their motivation to quit smoking. The results of the Intervention study showed that a statistically significant number of subjects observed an increase in their confidence and readiness to reduce or quit smoking one week after conversing with the chatbot.  Some subjects experienced a negative impact after conversing with the chatbot and more work should be done to investigate why these negative impacts are observed. |
| 18 | Block et al. (2015) | Diabetes prevention (including physical activity, eating habits, weight loss, stress, and sleep) | The aim was to evaluate the effectiveness of a fully automated algorithm-driven behavioral intervention for diabetes prevention, Alive-PD, delivered via the Web, Internet, mobile phone, and automated phone calls. | Health information (fasting glucose and lipids, HbA1c, height, body weight, waist circumference, and blood pressure), questionnaires (physical activity, diet), activity planning tool | Algorithm to automatically tailor and deliver print and phone messages to individuals | In intention-to-treat analyses, Alive-PD participants achieved significantly greater reductions than controls in fasting glucose, HbA1c, and body weight. Reductions in BMI, waist circumference, and TG/HDL were also significantly greater in Alive-PD participants than in the control group. At 6 months, the Alive-PD group reduced their Framingham 8-year diabetes risk from 16% to 11%, significantly more than the control group.  Participation and retention were good; intervention participants interacted with the program a median of 17 of 24 weeks and 71.1% were still interacting with the program in month 6.  As a fully automated system, the program has high potential for scalability and could potentially reach many of the 86 million US adults who have prediabetes as well as other at-risk groups. |
| 19 | Hudlicka (2013) | Mindfulness meditation | The objective of this research was to develop and evaluate a Virtual Mindfulness Coach for training and coaching in mindfulness meditation. | Questionnaires, conversations with embodied conversational agent. | Embodied conversational agent (ECA) to guide the student through training material and provide supportive coaching to help students establish a regular mindfulness practice.  ECA represented by a drawn image of a face (showing a variety of affective and conversational expressions - happy, sad, concerned, confused).  The verbal interaction is conducted via a mixed-initiative, natural-language, text-based dialog (free-form natural language input or multiple-choice based input).  Information about the student from questions asked, comments, and answers and frequency (on questions on a particular topic, positive vs. negative comments, student’s assessment of the overall experience, number of questions asked) analysed to adapt to the student’s knowledge and motivational state. | Findings from a pilot evaluation study indicate that the coach-based training is more effective in helping students establish a regular practice than self-administered training using written and audio materials.  The coached group also appeared to be in more advanced stages of change in terms of the transtheoretical model, and have a higher sense of self-efficacy regarding establishment of a regular mindfulness practice. These results suggest that virtual coach-based training of mindfulness is both feasible, and potentially more effective, than a self-administered program.  Of particular interest is the identification of the specific coach features that contribute to its effectiveness. Participants felt neutral to mildly positive about the coach’s ability to provide customized feedback and address questions or difficulties. They were slightly more positive about the coach’s ability to provide support and encouragement. The assessments of the coach’s ability to adapting non-verbal expressions to the student’s needs, and a feeling of ‘personal connection’ with the coach, were negative.  The study data suggest that the most successful coach feature was its ability to provide customized feedback and support, in an interactive manner. This was the case in spite of the negative assessments of the coach’s ability to conduct free-form, natural language dialog, and the negative assessment of the coach’s affective and social realism. |
| 20 | King et al. (2013) | Physical activity, sedentary behaviour | This first-generation feasibility study aimed to apply a behavioral science-informed user experience design (BSUED) process in developing smartphone applications to increase regular physical activity and decrease sedentary behavior (e.g., prolonged sitting) in adults who to date have received less attention in this field (i.e., midlife and older adults new to smartphone technology). | Accelerometer (built into smartphone), app data, questionnaires | Algorithm to inform the three physical activity apps to provide “just-in-time” feedback to users:  Analytic app: personalized and quantified goal-setting and behavioral feedback, problem-solving around barriers to behaviour change, and informational tips or advice for behavior change  Social app: real-time social normative feedback  Affect app: avatar whose movements and behaviors directly reflected the physical activity and sedentary levels of the user. | The results indicated that the three applications were sufficiently robust to significantly improve regular moderate-to-vigorous intensity physical activity and decrease leisure-time sitting during the 8-week behavioral adoption period.  Acceptability of the applications was confirmed in the post-intervention surveys for this sample of midlife and older adults new to smartphone technology.  Preliminary data exploring sustained use of the applications across a longer time period yielded promising results. The results support further systematic investigation of the efficacy of the applications for changing these key health-promoting behaviors. |
| 21 | Mohan (2020) | Physical activity, eating behaviour | Our research aims to develop intelligent collaborative agents that are human-aware - they can model, learn, and reason about their human partner’s physiological, cognitive, and affective states. In this paper, we study how adaptive coaching interactions can be designed to help people develop sustainable healthy behaviors. | App data (daily behavioural reports, judgements of target behaviour difficulty) | Rule-based algorithm:  PARCoach helps a trainee pick a relevant health goal, set an implementation intention, and track their behavior. During this process, the trainee identifies a specific goal-directed behavior as well as the situational context in which they will perform it. PARCcoach uses this information to send notifications to the trainee, reminding them of their chosen behavior and the context | Our results support the Common Model of Cognition (CMC) -based view of behavior change and demonstrate that the desiderata for proposed interactive system design is useful in producing behavior change.  Participants were able to use PARCcoach to select a target behavior and maintain a record of their successes and failures at achieving it every day.  Participants who selected target behaviors that they were confident in (behaviors were not difficult or hard) attempting were more likely to comply with it and consequently more likely to build a healthy habit. We observed a strong personal component and consequently, an ideal adaptive coach should be cognizant of difficulty as it pertains to each individual trainee.  CMC-based view suggests a novel strategy of reminding that relies on the CMC to retrieve the right goal at the right time. This is ensured by creating associations between elements of the environment context with a target behavior and strengthening the associations through reminders. We observed that such reminders are useful in improving behavioral compliance.  CMC-based view posits that difficulty of target behavior and reminding have additive effects on behavior compliance. This is because difficulty and reminding affect different aspects of the CMC. This was supported by our data.  Participants’ value judgements were influenced by their successes and failures at attempting the target behavior. Further, value judgements were predictive of behavior compliance. |
| 22 | Rahmani (2020) | Mental health management | We proposed the notion of Personalized Mental Health Navigation (MHN) as a goal-based cybernetics system allowing for a continuous cyclic loop of measurement, estimation, guidance, and influence to monitor and make sure that the person’s mental health state remains in a healthy zone. | Smartphones and wearable and portable sensors (sleep activity, physical activity, accelerometer, GPS etc), objective and subjective information (current mental state, context, lifestyle, adherence to guidance, prior mental/medical conditions experience, genetics) | Cybernetic feedback control system (including goals, monitoring, mental health estimation, personal models, and guidance components): for a continuous cyclic loop of measurement, estimation, guidance, and influence to monitor and make sure that the person’s mental health state remains in a healthy zone.  Goals: to capture, store, and keep track of the desired mental states and goals of the user to better inform the estimation and later the guidance procedures  Monitoring: multimodal stream of information is collected from an individual to provide a holistic picture of an individual’s status augmenting the traditional monitoring and therapy methods  Mental health estimation: access a range of other personal data, including prior mental/medical conditions experienced by the individual and their genetics to build and update models of physiological, psychological, and behavioral patterns for each individual to personalize the monitoring, estimation, and guidance provided by the system.  Guidance: interventions which depending on their types and risk-levels can be provided automatically using AI-based recommender systems | Pilot data and the case study provide evidence for the feasibility of the monitoring and estimation components of the MHN model, where objective and subjective data were able to be collected in real-time (allowing for capturing of the physical and mental states of an individual while also accessing this data to estimate their well-being (i.e., shifts in their sleep and emotions)).  Pilot data captured monitoring, estimation components. The guidance component may be incorporated as part of an intervention in future studies. |
| 23 | Sillice et al. (2018) | Physical activity, sun protection behaviours | The purpose of this study was to examine participants’ experiences using Relational Agents (RA) to increase participant engagement and promote behavior change *.* | Demographic information, conversations with RA | Expert system intervention (through RA): to provide participants with unique matched information and intervention based on their health risks and attitudes toward exercise and sun protection  RA: maintained a conversational approach with participants throughout the program, while providing them with support and encouragement to engage in regular exercise and sun protection behaviors and eventually maintenance. It uses speech, gaze, hand gesture, intonation, and other nonverbal modalities to emulate the experience of human face-to-face conversation with their users | Participants were motivated by, and satisfied with, the intervention.  Participants viewed the RA as supportive, informative, caring, and reported positive behavior change in both exercise and sun protection. Some participants noted the RA was less judgmental and less “overbearing” compared with a human counselor; other participants said that the interaction was sometimes repetitive or overly general. The majority of participants viewed the RA as an important contributor to their behaviour change for exercise, sun protection, or both.  RAs provide an innovative and attractive platform to increase exercise and sun protection behaviors and potentially other health behaviors. |
| 24 | Traficante (2004) | Physical activity | To test:  (1) preference for computer-based interventions versus treatment delivered by a human on the telephone and (2) the effectiveness of these two treatments as compared to each other and to a control group of similar characteristics. | Physical activity log (daily exercise), questionnaires (stages of change, decisional balance, self-efficacy) | Expert system to generate personalized feedback reports | The data on 263 participants showed that significantly more males than females preferred the computer generated, personalized reports from an expert system via print.  Regardless of preference, 88% of the sample felt they would remain an active participant in the study even if they received information in a manner they did not prefer.  By the six-month follow-up the group receiving computer-generated reports through the mail was exercising 120.3 minutes per week versus 101.3 minutes for the telephone participants. Although there was no statistical difference between these two groups, the results were significantly better than the control group, which averaged only 46.4 minutes of exercise per week.  The results of this study show that individualized feedback from an expert system, regardless of the means of delivery and participant preferences, produced significant increases in physical activity when compared to a control group. |
| 25 | Galvão Gomes da Silva et al. (2018) | Physical activity | The aim of this study was to explore participants’ qualitative experiences of a motivational interview delivered by a social robot, including their evaluation of usability of the robot during the interaction and its impact on their motivation. | Conversations with NAO robot | NAO robots (humanoid, child-sized social robots): programmed with Choregraphe software to deliver a scripted motivational interview focused on increasing physical activity. | Participants found the instructions clear and the navigation easy to use.  Most enjoyed the interaction but also found it was restricted by the lack of individualized response from the robot.  Many positively appraised the nonjudgmental aspect of the interview and how it gave space to articulate their motivation for change.  Some participants felt that the intervention increased their physical activity levels.  Social robots can achieve a fundamental objective of motivational interviewing, encouraging participants to articulate their goals and dilemmas aloud. Because they are perceived as nonjudgmental, robots may have advantages over more humanoid avatars for delivering virtual support for behavioral change. |
| 26 | Maimone et al. (2018) | Physical activity and eating behaviour (in workplace) | To present a general purpose architecture for persuasion scenarios and behavioral change. | Food diary, pedometers, GPS | Rule-based reasoning system: to send personalized and contextual motivational messages (textual or graphical) to the employee to give feedback on acquired/provided data, suggestions and guidelines for an healthy behavior. Weekly challenges and weekly report are also generated to allow employees to increase awareness on their lifestyle | Interviews were conducted to evaluate the impact of the mobile application in the routine at work setting and extra-work setting, at the end of the seven weeks of pilot study.  In general users appreciated the system and considered the mobile application a useful tool, especially for increasing the awareness about their eating habits. Indeed, users provided mainly dietary data because few of them had pedometer bracelets. |
| 27 | Martin et al. (2015) | Physical activity | To test the hypothesis that a fully automated, fully mobile, and physician-designed mHealth intervention using new technologies to provide individual encouragement and foster feedback loops increases physical activity | Smartphone, Fitbug Orb (wearable, display-free, triaxial accelerometer that pairs with low-energy Bluetooth with compatible smartphones), questionnaire (personal and clinical characteristics) | Tracking component: transmits continuous physical activity information to participants  Texting component: algorithm used to send automated, personalized messages to participants three times a day. | An automated tracking-texting intervention increased physical activity with, but not without, the texting component. Coupling smart texts with activity tracking led to the best physical activity outcomes, with nearly twice as many participants in the text-receiving arm achieved the 10 000 steps/day goal compared with the other groups.  Aerobic activity levels were disproportionately low compared with steps and total activity time, but appeared most responsive to intervention.  The mActive trial lends support to the notion of new mHealth devices as facilitators, not drivers, of behavior change because sequential randomization suggested that unblinding to device data did not significantly modify behavior, whereas coupling it with smart texts did. |
| 28 | Stein & Brooks (2017) | Weight loss | The objective of this study was to evaluate weight loss, changes in meal quality, and app acceptability among users of the Lark Weight Loss Health Coach AI (HCAI), with the overarching goal of increasing access to compassionate health care via mobile health. | Demographic information, App data (weight loss, meal quality, physical activity, sleep data, duration and amount of app use), Smartphone sensors (sleep and physical activity), Survey (user trust survey to assess app usability and acceptability) | Artificial intelligence health coach responds to users’ specific input, such as food and beverage consumption, weight, or sleep duration, with relevant content such as praise, educational material, or reflection. | Weight loss was 2.38% of baseline weight. The average duration of app use was 15 weeks, and users averaged 103 sessions each. Predictors of weight loss included duration of AI use, number of counselling sessions, and number of meals logged.  Percentage of healthy meals increased by 31%. The in-app user trust survey had a 100% response rate and positive results, with a satisfaction score of 87 out of 100 and net promoter score of 47.  This study showed that use of an AI health coach is associated with weight loss comparable to in-person lifestyle interventions. It can also encourage behavior changes and have high user acceptability. |
| 29 | Stephens et al. (2019) | Weight management | This study assessed the feasibility of integrating a behavioural coaching chatbot (Tess) in behavioral counseling of adolescent patients coping with weight management and prediabetes symptoms. | Goals, Conversations with chatbot | Behavioural coaching AI chatbot (Tess):  A combination of technologies, algorithms, and machine-learning strategies: enables a range of features that are customizable.  Emotion algorithm that enables analyzation of the incoming messages from users based on the emotions expressed. | Adolescent patients reported experiencing positive progress toward their goals 81% of the time. The large number of messages (4,123) exchanged and patients’ reported usefulness ratings (96% of the time) illustrate that adolescents engaged with and viewed this chatbot as helpful.  Conversations varied in length, with some as short as a few seconds to longer than an hour, which differs from usual treatment that has a set, prescribed duration.  These results highlight the feasibility and benefit of support through artificial intelligence, specifically in a pediatric setting, which could be scaled to serve larger groups of patients. |
